# Characteristics of interstitial lung disease in patients from post-marketing data on metastatic breast cancer patients who received abemaciclib in Japan

**DOI:** 10.1007/s12282-020-01207-8

**Published:** 2021-01-16

**Authors:** Yucherng Chen, Satoshi Noma, Yoshio Taguchi, Masashi Takahashi, Junji Tsurutani, Shiho Mori, Sachi Sakaguchi, Hiroya Asou, Keisuke Tomii

**Affiliations:** 1 Eli Lilly Japan K.K, Lilly Plaza One Building, 5-1-28 Isogamidori, Chuo-Ku, Kobe, Hyogo 651-0086 Japan; 2 Department of Radiology, Tenri Hospital, Tenri, Japan; 3 Department of Respiratory Medicine, Tenri Hospital, Tenri, Japan; 4 Yujin Yamazaki Hospital, Hikone, Japan; 5 Advanced Cancer Translational Research Institute, Showa University, Tokyo, Japan; 6 Eli Lilly Japan K.K, Tokyo, Japan; 7 Kobe City Medical Center General Hospital, Kobe, Japan

**Keywords:** Abemaciclib, Breast cancer, Cyclin-dependent kinase 4/6, Interstitial lung disease

## Abstract

**Background:**

This study evaluated characteristics of patients treated with abemaciclib and diagnosed with interstitial lung disease (ILD), using 12-month post-marketing data from the real-world setting in Japan.

**Methods:**

Spontaneous reports of adverse events in patients receiving abemaciclib were collected regularly from healthcare providers (HCPs) from November 30, 2018, to November 29, 2019. Detailed follow-up was requested on suspected ILD cases via questionnaires and/or interviews. Radiological images (when available) were reviewed by an ILD adjudication committee of specialists. The age distribution of patients prescribed abemaciclib in Japan was estimated based on insurance claims data.

**Results:**

Of 4700 patients estimated to be exposed to abemaciclib, 82 cases of ILD were reported (46 serious, 13 fatal). Most (91%) had ≥ 1 symptom at diagnosis, commonly dyspnea/shortness of breath (59%), cough (44%), and/or fever (37%). The majority (68%) received steroid therapy (24 [56%] recovered/recovering; 5 [12%] not recovered; 13 [30%] deaths, 1 [2.3%] unknown). No specific imaging patterns or sites of predilection were identified, but a diffuse alveolar damage (DAD) pattern was observed at outcome in 3 of 4 evaluated fatal cases (16 in total evaluated). Features of fatal cases included advanced age, pre-existing interstitial change, and advanced Eastern Cooperative Oncology Group Performance Status.

**Conclusion:**

Advanced age and a DAD pattern were identified as potential risk factors for cases with poorer outcomes, as previously reported for drug-induced ILD. HCPs should consider the benefit–risk profile when prescribing abemaciclib, informing patients of risks and regularly monitoring treated patients to ensure early detection and treatment of ILD.

**Supplementary Information:**

The online version contains supplementary material available at 10.1007/s12282-020-01207-8.

## Introduction

Abemaciclib is a selective cyclin-dependent kinase 4/6 (CDK4/6) inhibitor used in the management of hormone receptor positive (HR +), human epidermal growth factor receptor 2 negative (HER2-) unresectable or recurrent breast cancer. In the phase 3 clinical trials MONARCH 2 and MONARCH 3, abemaciclib significantly improved outcomes in patients with HR + , HER2- advanced breast cancer in combination with an aromatase inhibitor as initial therapy [1] or with fulvestrant after progression on endocrine therapy [2, 3]. Abemaciclib was approved for use in Japan in September 2018, followed by product launch in November 2018.

Interstitial lung disease (ILD) is a collection of potentially fatal inflammatory lung disorders that result in fibrotic lesions within the lung interstitium. ILD develops from various causes [4]. In particular, ILD is a well-recognized potential complication of many different therapeutic agents [5], with drug-induced ILD accounting for an estimated 3–5% of prevalent cases of ILD [5]. Anti-cancer agents are the most common causes of drug-induced ILD [5, 6]. Reported incidence of anti-cancer agent-induced ILD varies in the literature (e.g., bleomycin, 6.8–15%; everolimus, 2.8–58.0%; erlotinib, 0.9–5.9%; gefitinib: 1.9–3.5%) [5], and the reported incidence has been noted as higher in Japan than in other countries [5–7].

Data have emerged suggesting ILD is a class side effect of CDK4/6 inhibitors [8]. Currently, ILD is included as a clinically significant adverse drug reaction (ADR) in the package insert for abemaciclib in Japan with a frequency of 2.0% [9], as identified by standardized Medical Dictionary for Regulatory Activities (MedDRA, version 19.1) queries (SMQs) for ILD of the manufacturer’s safety databases. The frequency of death due to ILD in the New Drug Application in Japan was 0.2% (n = 1 of 441) and 0.3% (n = 1 of 327), from the MONARCH 2 and MONARCH 3 clinical trials, respectively. However, at the time of product launch, the risk of developing ILD after receiving abemaciclib for Japanese patients in the real-world setting was not known.

Early-phase post-marketing vigilance (EPPV) is a post-marketing safety monitoring process in Japan, which promotes the appropriate use of the product during the first 6 months after product launch. During the 6-month EPPV period for abemaciclib, 14 cases of serious ILD were reported, 3 of which were fatal. In May 2019, these findings resulted in a ‘Dear Healthcare Professionals’ (DHCP) Letter of Rapid Safety Communication (a ‘Blue Letter’) from the Pharmaceuticals and Medical Devices Agency (PMDA) and Ministry of Health, Labor, and Welfare notifying HCPs in Japan of the risk of ILD with abemaciclib treatment [10]. Following consultation with the PMDA, the manufacturer extended the duration of monitoring for ILD to up to 12-month post-launch. The objective of the EPPV and additional 6-month monitoring program was to gain a better understanding of the real-world safety of abemaciclib. Using post-marketing data from clinical practice in Japan during this 12-month monitoring period, we report here the clinical and imaging characteristics of patients treated with abemaciclib and diagnosed with ILD.

## Patients and methods

### Patients

Data were collected on all ILD cases initially reported between November 30, 2018, and November 29, 2019, in patients being treated with abemaciclib in clinical practice in Japan. In total, 988 hospitals and clinics purchased abemaciclib for use in patients in Japan during this period. Radiological images were provided with the consent of the patients (or their families) via their HCPs, in line with institutional processes.

### Data collection

The data in this report are based on spontaneous reports of adverse events (AEs) by HCPs and patients. During the EPPV period, pharmaceutical representatives contacted HCPs at healthcare facilities prescribing abemaciclib to report if any AEs occurred during abemaciclib treatment. HCPs were contacted at a minimum every 2 weeks for the first 2 months and then at a minimum of monthly thereafter until 6 months post launch. If any potential AEs were reported by HCPs, the pharmaceutical representative obtained additional information about the AE and sent a report to the manufacturer, Eli Lilly Japan K.K. Following the EPPV period, reports of ILD were collected for an additional 6-month monitoring period. Collection methodology was the same for both periods, and data on ILD cases from the EPPV period and additional 6-month monitoring period were combined. Cases were excluded from analysis if additional information from HCPs confirmed the case was not ILD; otherwise, all reported cases of ILD or suspected ILD were included in the analysis of this study.

For AEs of ILD or suspected ILD reported by either HCPs or patients, Eli Lilly Japan K.K requested follow-up information using a specific questionnaire form to capture demographic and clinical information (patient characteristics, past medical history, treatment history, ILD event, ILD treatment, associated laboratory test data, imaging data). In cases that were serious and/or resulted in death, chest X-ray or computed tomography (CT) scans and details of disease course were requested and, if consent from the HCP was obtained, in-person interviews of HCPs were conducted. AEs of ILD in this report were deemed ADRs (i.e., AEs considered to have a causal relationship with treatment).

### Data processing and analysis

HCP-reported terms were entered into the manufacturer’s electronic safety database and coded to MedDRA, Version 22.1. The safety database was searched using the SMQ for ILD to identify ADRs that were ILD or could potentially be diagnosed as ILD (SMQ ILD preferred terms are listed in Supplementary Table 1). For all such cases identified as ILD, additional data, as available, were derived from information in the case narratives provided by the HCPs. ILD case data are reported as of the date of the first report, with information on case seriousness and outcome (e.g., serious [fatal], serious [non-fatal], and non-serious cases) updated until the data cut-off date of November 29, 2019. Seriousness of each ILD case was assessed as reported by the reporting HCP. In cases where seriousness was not provided by the reporting HCP, seriousness was determined by Eli Lilly Japan K.K. based on standard criteria as specified in the International Council for Harmonisation of Technical Requirements for Pharmaceuticals for Human Use (ICH) guidelines. ICH guidelines specify any AE is serious if it is life-threatening or results in death, inpatient hospitalization, prolongation of existing hospitalization, persistent or significant disability or incapacity, congenital anomaly, or other medically important condition [11]. The timing of ILD onset was defined as the period between the date of first abemaciclib dose and the date on which imaging was first conducted to evaluate or confirm suspected ILD. Symptoms reported were based on medical judgement by HCPs. ILD cases by age category were tallied according to the age at which the first symptom was observed or as of ILD diagnosis, whichever came first.

#### Calculations of number exposed

Estimates of the number of patients exposed were calculated by dividing the total number of milligrams sold (based on the Japanese component of global sales data for abemaciclib) by the estimated monthly dose of abemaciclib per patient (based on the combination of dosing information from abemaciclib registration clinical trials and the distribution of patients across dosing strategies for abemaciclib):

Total abemaciclib sold (mg)/estimated monthly dose per patient (mg) = total months of therapy.

This figure (total months of therapy) was then divided by the average months of therapy for an individual patient (based upon available data for abemaciclib over the time period of this analysis) to determine the estimated number of patients exposed:

Total months of therapy/average duration of therapy in months per patient = number of patients treated with abemaciclib.

#### Estimation of the age distribution of the background population of patients in Japan prescribed abemaciclib

The age distribution of the background population of patients who received abemaciclib during the study period in Japan was estimated based on insurance claims data in the Medical Data Vision (MDV) database [12]. The study population consisted of adult (aged ≥ 18 years) patients who made insurance claims for abemaciclib treatment (at least 1 claim) between December 1, 2018 (following product launch) and September 30, 2019 (the end of data availability). The follow-up period was defined as the entire period that spanned from the date of the first prescription for abemaciclib (not inclusive) until the earliest of the following events: (1) treatment gap: patients were censored if abemaciclib was not dispensed for more than 28 days after exhaustion of the last dispensed supply and were followed until the last day with drug supply on-hand prior to a > 28-day gap in therapy; (2) treatment switch: patients were followed until the date of discontinuation (defined as 1 day before initiating a new regimen that does not contain the abemaciclib). Patients who remained on abemaciclib but changed to a different endocrine therapy partner had continued to follow up; (3) death (all causality); or (4) end of the data availability (September 30, 2019).

### Imaging

Imaging from selected cases was reviewed by an ILD adjudication committee (ILDAC), consisting of 4 independent specialists (2 radiologists [M.T. and S.N.] and 2 pulmonologists; K.T. and J.T]) who had been established by Eli Lilly Japan to monitor and evaluate ILD during the clinical trial and post-marketing phases of drug development. There were no systematic criteria for the selection of cases, and not all cases were reviewed. Cases were generally examined in the order of acquisition from the hospitals, due to time constraints, with prioritization of cases with sufficient information (in addition to imaging data) for full assessment. Fatal cases were also prioritized over non-fatal cases. ILD diagnoses were confirmed by comprehensive review of patient narratives, laboratory and clinical investigations (e.g., echocardiography, bronchoalveolar lavage), and imaging from CT or chest X-rays, obtained when available from the healthcare facilities.

## Results

### Estimated abemaciclib exposure and ILD frequency

**Fig. 1:**
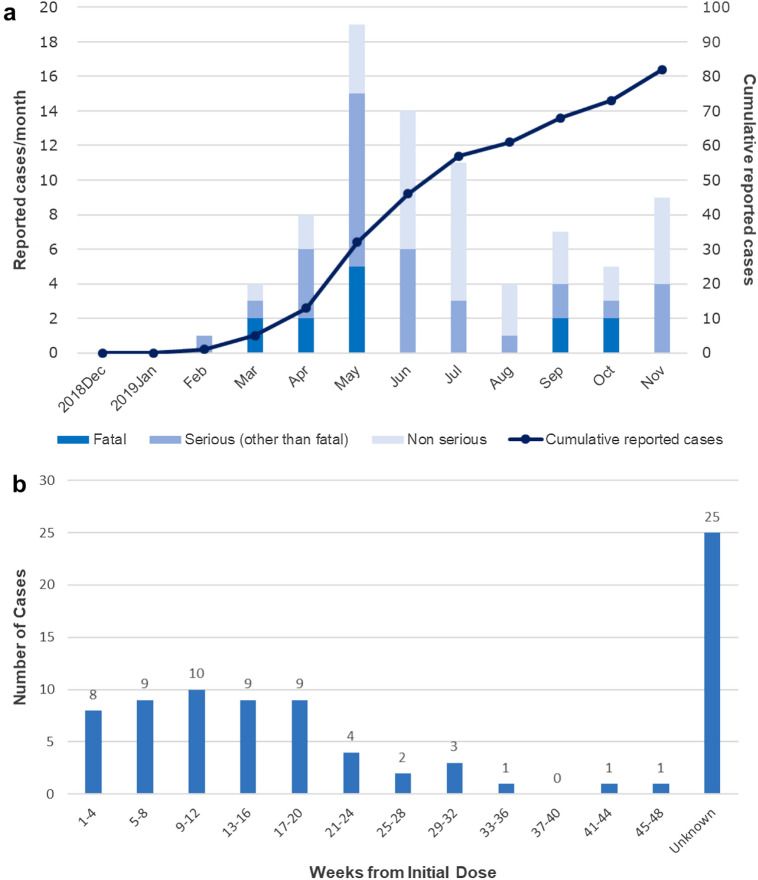
Summary of reports of ILD in patients treated with abemaciclib. Data are shown by **a** ILD reports per month by seriousness; and **b** ILD onset, representing the time from the first dose of abemaciclib to first imaging study for ILD. The line in (**a**) shows the cumulative cases of ILD across the monitoring period. ILD, interstitial lung disease

During the first year after the launch of abemaciclib in Japan, approximately 4700 patients were exposed to abemaciclib, and ILD was reported for 82 patients (85 events) (82 of 4700; 1.7%) in Japan. Of these, 13 cases (16%) were fatal, 33 (40%) were serious but not fatal, and 36 (44%) were non-serious. The overall frequency of death due to ILD in abemaciclib-treated patients was 0.3% (13 of 4700). The reporting of ILD per month is shown in Fig. 1a. After the issuance of the Blue Letter on May 17, 2019, the number of ILD reports temporarily surged, with more non-serious cases reported.

### Timing of ILD onset

The timing of ILD onset varied (Fig. 1b). Among cases with available information on ILD onset (n = 57 of 82; 70%), onset was most frequently reported within 5 months from the first dose of abemaciclib (n = 45 of 57 cases; 79%). However, 12 cases (21%) had onset ≥ 6 months from the initiation of abemaciclib treatment (Fig. 1b). For 25 cases (30%), information was not available on the date of first abemaciclib dose and/or imaging after ILD was suspected, and onset is considered ‘not reported’ for these cases.

### Symptoms

Among 82 cases, information regarding initial symptoms at diagnosis was reported in 70 cases. Of these, 64 (91%) had at least 1 symptom at the time of ILD diagnosis. In addition, 5 cases (7%) were asymptomatic, and diagnosis was made from imaging data. One additional case had no cough or fever, but the presence or absence of other symptoms was unknown. The most common symptoms at diagnoses were dyspnea/shortness of breath (n = 41 cases; 59%), cough (n = 31 cases; 44%), fever (n = 26 cases; 37%), and malaise/fatigue (n = 22 cases; 31%). Only 6 (9%) patients exhibited all 3 of the most common symptoms together (fever, cough, and dyspnea/shortness of breath). Of these, fever and dyspnea/shortness of breath were reported in a higher proportion of fatal cases than non-fatal cases (serious and non-serious) (Table 1).

**Table 1:** Common ILD symptoms by seriousness

Symptom	Fatal (N)			Serious (excluding fatal) (N)			Nonserious (N)			Total (N)		
	Yes	No	Unk	Yes	No	Unk	Yes	No	Unk	Yes	No	Unk
Fever	7	1	5	14	6	13	5	9	22	26	16	40
Dyspnea/Shortness of breath	8	1	4	19	5	9	14	5	17	41	11	30
Cough	5	2	6	12	9	12	14	6	16	31	17	34

Seriousness was defined by standard International Council for Harmonisation of Technical Requirements for Pharmaceuticals for Human Use criteria as any symptom judged by the healthcare provider or study sponsor as being life-threatening or resulting in death, inpatient hospitalization, prolongation of existing hospitalization, persistent or significant disability or incapacity, congenital anomaly, or other medically important condition

ILD, interstitial lung disease; Unk, unknown

### Treatment and outcomes

**Fig. 2:**
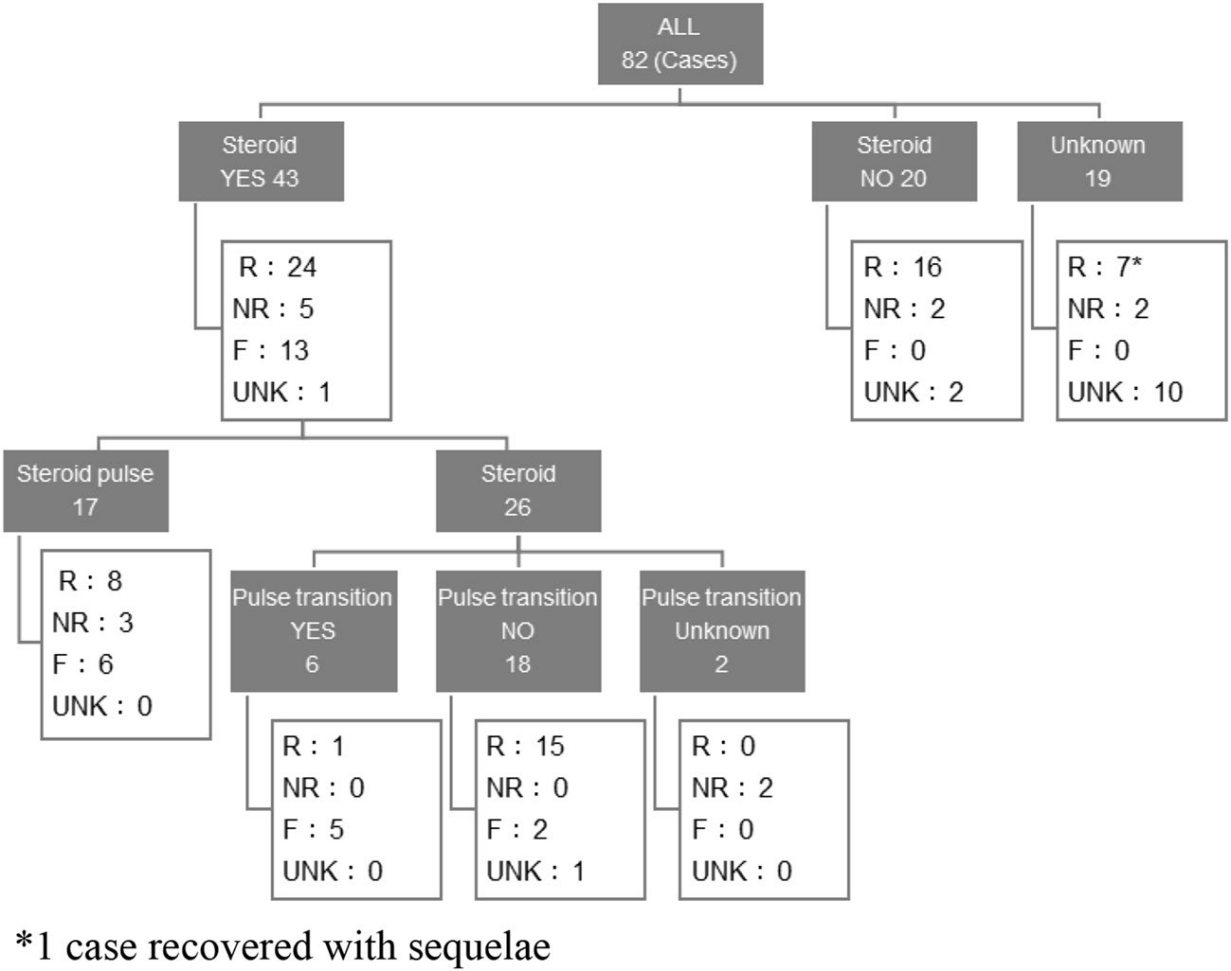
Outcomes for ILD cases with and without steroid treatment. Steroid pulse treatment included ‘mini-pulse’ treatment (≥ 0.5 g/day of intraveneous methylprednisolone). Pulse transition refers to patients who started on non-pulse steroid therapy but were switched to pulse therapy. F, fatal; ILD, interstitial lung disease; NR, not recovered; R, recovered/recovering; UNK, unknown

Abemaciclib was discontinued in all cases of ILD where information was available (n = 75 known; n = 7 unknown). Information regarding treatment with steroids was available for 63 cases, with 43 (68%) receiving steroid therapy and 20 (32%) not receiving steroid therapy. Of those receiving steroid therapy, 23 (53%) received pulse therapy or transitioned to pulse therapy after initially receiving non-pulse steroid therapy. Outcomes of treatment are shown in Fig. 2. Among the 43 patients who received steroid therapy, 24 (56%) were recovered/recovering, 5 (12%) were not recovered, and 13 (30%) had died (n = 1 unknown outcome). Of the 23 patients who received pulse/pulse transition therapy, 9 (39%) were recovered/recovering, 3 (13%) were not recovered, and 11 (48%) had died. Among the 20 patients known to have not received steroid therapy, 16 (80%) recovered/recovering, all of which were non-serious cases, and 2 (10%) had not recovered (n = 2 unknown outcome) (Fig. 2).

### ILD cases by age and seriousness

**Fig. 3:**
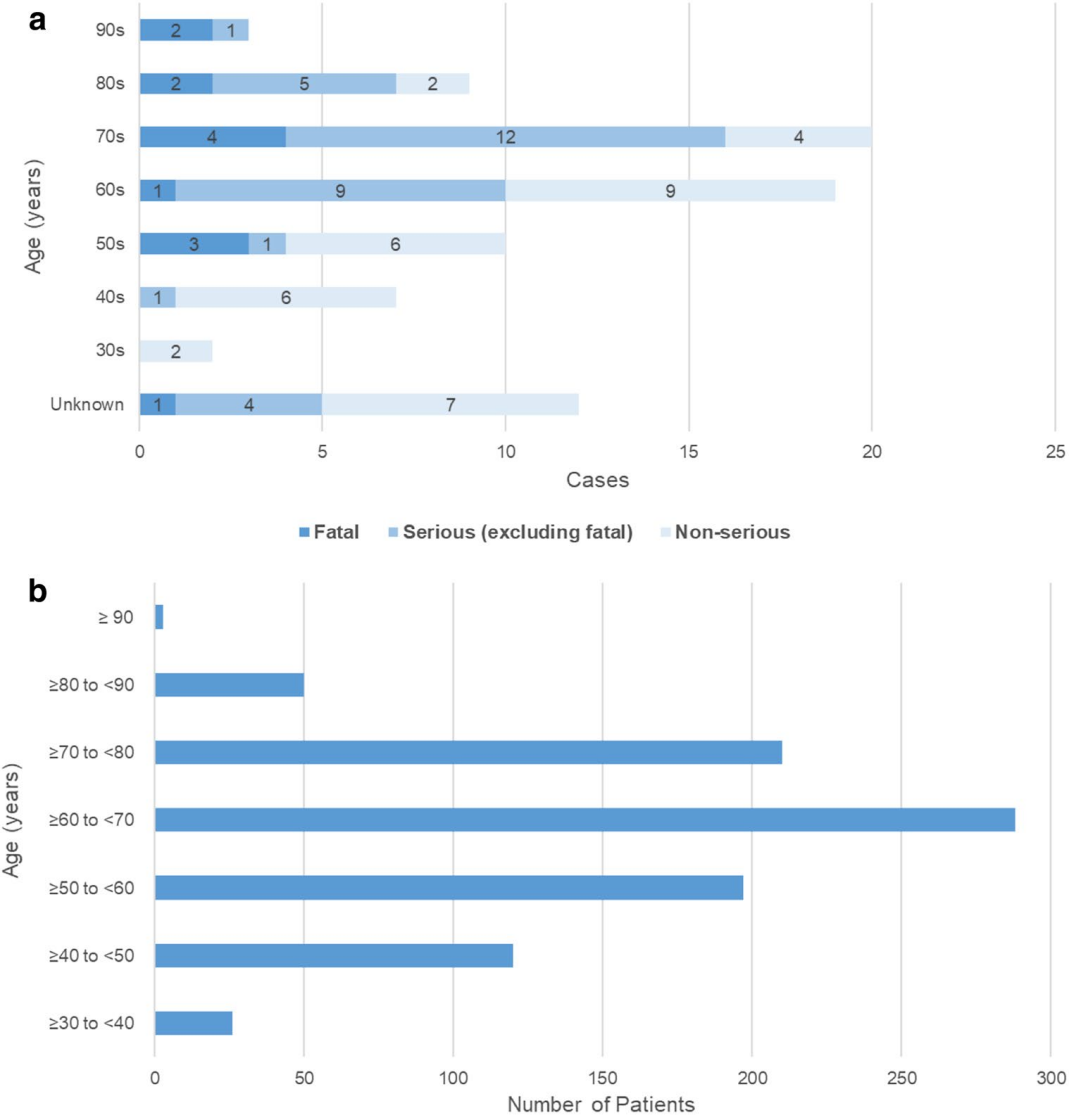
Age distribution of ILD cases and the background population of patients prescribed abemaciclib in Japan. **a** ILD cases by age and seriousness; **b** age distribution of abemaciclib-prescribed patients in Japan from insurance claims database

Information regarding the age of patients was available for 70 ILD cases (n = 12 age unknown) (Fig. 3a). Of these, 32 (46%) were aged ≥ 70 years, including 20 (29%), 9 (13%), and 3 (4%) aged between 70–79 years, 80–89 years, and 90–99 years, respectively. In patients aged < 70 years, there were 38 cases of ILD, of which 4 (11%) were fatal, 11 (29%) were serious, non-fatal, and 23 (61%) were non-serious cases. In comparison, in patients aged ≥ 70 years, there was a trend toward more serious and fatal cases (n = 32 total cases: 8 [25%] fatal cases, 18 [56%] serious cases; 6 [19%] non-serious cases) (Fig. 3a). To provide context for the frequency of ILD cases observed at different age ranges, the age distribution of patients in Japan who were prescribed abemaciclib was estimated based on insurance claims data (Fig. 3b). Of 894 patients who made insurance claims for abemaciclib treatment from December 1, 2018 to September 30, 2019, 263 (29%) were aged ≥ 70 years, including 210 (23%), 50 (6%), and 3 (0.3%) aged between 70–79 years, 80–89 years, and 90–99 years, respectively. Based on this overall age distribution of the background population in Japan, more ILD cases occurred with increasing age (i.e., patients aged ≥ 70 years comprised 46% of ILD cases but only 29% of the background population).

### Fatal cases

Features of the fatal cases are summarized in Table 2. Nine of the fatal cases (69%) had at least 1 of the following: advanced age (≥ 70 years old), pre-existing interstitial change, or advanced Eastern Cooperative Oncology Group Performance Status (ECOG PS) ≥ 2. Specifically, 8 fatal cases (62%) were patients aged ≥ 70 years (4 cases [31%] < 70 years of age; 1 unknown). In addition, 4 fatal cases (31%) had pre-existing changes in the lung interstitium and 4 (31%) cases had an ECOG PS ≥ 2 at the start of abemaciclib treatment. Five of the 13 fatal cases (38%) had received prior CDK4/6 inhibitor treatment (Table 2).

**Table 2:** Clinical characteristics of the 13 fatal ILD cases

Characteristic	Cases (n)
Age when diagnosed	
50–59 years	3
60–69 years	1
70–79 years	4
80–89 years	2
90–99 years	2
Unknown	1
Pre-existing interstitial change	
Lung MAC infection with interstitial change^a^	1
Interstitial pneumonia with autoimmune features	1
Radiation pneumonitis	1
Slight interstitial change at bilateral posterior lower lobe	1
ECOG PS (at abemaciclib start)	
0	5
0–1 (including cases > 0 and ≤ 1)	3
1–2 (including cases > 1 and ≤ 2)	2
3	2
Unknown	1
Prior CDK4/6 inhibitor	
Yes	5
No	6
Unknown	2
Other	
Carcinomatous lymphangitis	1
Re-administration of abemaciclib^a^	1
Chest CT finding^b^	
DAD^c^	3
Non-DAD (OP-like)^d^	1
Not evaluated	9

CDK4/6, cyclin-dependent kinase 4/6; DAD, diffuse alveolar damage; ECOG PS, Eastern Cooperative Oncology Group Performance Status; ILDAC, interstial lung disease adjudication committee; MAC, Mycobacterium avium complex; n, number of cases in category; OP, organizing pneumonia

^a^Same case

^b^Classification of cases is by the imaging pattern most representative of the case, as evaluated by the ILDAC

^c^1 case that initially showed Faint GGO but later developed into DAD

^d^Includes 1 case that initially showed an OP pattern that later developed mixed features of OP/DAD but did not progress fully to DAD

### CT imaging patterns

**Fig. 4:**
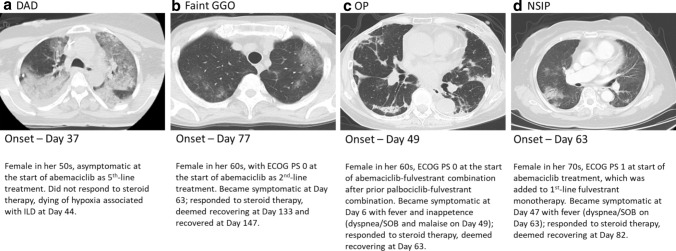
Radiological findings for ILD in patients treated with abemaciclib. Representative CT images from 4 individual cases which were diagnosed as ILD in patients treated with abemaciclib by the ILD adjudication committee. Days are from first abemaciclib dose. **a** DAD image pattern showing extensive consolidation in the lower lobe on both sides, which was surrounded by GGO that occupied two-thirds of the lower lobe on both sides. **b** Non-DAD, faint GGO image pattern showing faint GGO in the whole lung field, which was dominant at the periphery. **c** OP image pattern beneath pleura at bilateral lower lobe. **d** NSIP pattern showing an interstitial shadow. CT, computed tomography; DAD, diffuse alveolar damage; ECOG PS, Eastern Cooperative Oncology Group Performance Status; GGO, ground glass opacity; ILD, interstitial lung disease; N, number of cases; NSIP, non-specific interstitial pneumonia; OP, organizing pneumonia. SOB, shortness of breath

Imaging data from 16 cases were evaluated by the ILDAC. A breakdown of imaging patterns by seriousness is shown in Table 3. Four patterns were observed for patients treated with abemaciclib and diagnosed with ILD: imaging patterns had features consistent with a diffuse alveolar damage (DAD) pattern in 4 cases, an organizing pneumonia (OP) pattern in 4 cases, a faint ground glass opacity (GGO) pattern in 3 cases, and a non-specific interstitial pneumonia (NSIP) pattern in 1 case (4 cases not classified). The DAD pattern was the predominant imaging pattern observed at the time of outcome in fatal cases (Table 2). Representative CT patterns are shown in Fig. 4.

**Table 3:** CT imaging patterns by seriousness of the 16 ILD cases reviewed by the ILDAC

Chest CT scan Imaging pattern^a^	Fatal (N)	Serious (excluding Fatal) (N)	Nonserious (N)
DAD	3^b^	1	0
Non-DAD	1	5	2
OP-like	1^c^	2	1
Faint GGO	0	2	1
NSIP-like	0	1	0
Other (indeterminable etc.)	0	3	1

CT, computed tomography; DAD, diffuse alveolar damage; GGO, ground glass opacity; ILD, interstitial lung disease; ILDAC; ILD adjudication committee; N, number of cases; NSIP, non-specific interstitial pneumonia; OP, organizing pneumonia

^a^Classification of cases is by the imaging pattern most representative of the case, as evaluated by the ILDAC

^b^Includes a case that initially showed faint GGO but turned into DAD

^c^Case that initially showed an OP pattern that later developed mixed features of OP/DAD but did not progress fully to DAD

## Discussion

Given its potentially serious course and fatal outcome, a better understanding of ILD in abemaciclib-treated patients is required for prompt and accurate diagnosis. To gain insight into the characteristics of patients receiving abemaciclib who were diagnosed with ILD in the real-world setting in Japan, this analysis evaluated post-marketing data collected from abemaciclib-treated patients during the first 12 months after product launch. In this setting, the estimated incidence of ILD was 1.7% with 0.3% fatality. This estimated real-world incidence of patients receiving abemaciclib who are diagnosed with ILD is in accordance with the ILD incidence reported on the Japanese label for abemaciclib in clinical trials (2.0%) [9]. The incidence of patients receiving abemaciclib who were diagnosed with ILD is also broadly comparable to incidence rates from ILD risk assessments conducted in clinical settings in Japan for other common anti-cancer agents known to cause ILD [13, 14], although ILD incidence rate estimates can vary widely in the literature (e.g., 3–58% in patients treated with everolimus) [5, 15]. Similarly, the mortality rate among ILD cases in the current study (13 of 82; 16%) is comparable to that observed for many other anti-cancer therapies, although mortality rates also vary in the literature (e.g., 5–20% for everolimus) [5].

This study has several limitations due to the nature of post-marketing monitoring, which restricts the comprehensiveness of the data and their interpretation. Notably, data were gathered only for cases of ILD, and no direct information is available for the underlying patient population, which prevents a more detailed assessment of factors that may increase the risk of ILD development. It should also be acknowledged some cases of ILD may not have been reported by the HCPs and the MDV database may not have captured all abemaciclib-treated patients in Japan. With these limitations in mind, this analysis identified potential features of ILD in patients treated with abemaciclib, which will require further observation to confirm.

No clear pattern of ILD onset emerged from the current data. Although most cases were reported within 5 months of the first dose of abemaciclib, the onset of ILD varied widely, from acute onset (i.e., within days) to 11 months after the first dose of abemaciclib. Given this analysis was conducted only 12 months after product launch, these data could also potentially be confounded in that the number of long-term treated patients is unknown, and a smaller number of patients could have used abemaciclib for > 5 months compared to < 5 months. In addition, no clear pattern of ILD symptomology emerged for patients who received abemaciclib. Although nearly all patients who were diagnosed with ILD exhibited at least 1 symptom of ILD at the time of diagnosis, respiratory symptoms were not evident in all patients, and 5 patients were asymptomatic. Further complicating the identification of ILD cases in patients treated with abemaciclib in the real-world setting, the most frequently reported symptoms were similar to the common symptoms of other types of respiratory diseases (i.e., dyspnea/shortness of breath, cough, and fever). This lack of a predictable onset period and specific symptomology for ILD in patients treated with abemaciclib highlight the need for continuous monitoring for ILD for as long as the patient is treated with abemaciclib to ensure patient safety. Furthermore, regular monitoring should include careful physical examination and checks of vital signs, including assessment of peripheral capillary oxygen saturation.

Of the patients not treated with steroid therapy, over three-quarters were reported as recovered or recovering at the data cut-off date, all of which were non-serious cases. This suggests non-serious ILD in patients treated with abemaciclib could be managed by abemaciclib discontinuation alone. However, the majority (72%) of patients with ILD required steroid therapy, and although many (57%) responded to treatment, some did not and died (30%). Importantly, of the patients who died, over two-third had risk factors associated with potentially underlying or complicating ILD, including pre-existing interstitial changes within the lungs, advanced age (≥ 70 years old), and/or advanced ECOG PS (≥ 2). Although it is yet to be determined that these clinicopathological features predict the risk of ILD in patients treated with abemaciclib, pre-existing interstitial lesions and advanced age are known risk factors for developing drug-induced ILD [13], and higher ECOG PS scores predispose patients to worse outcomes. In addition, 38% had received prior CDK4/6 inhibitor therapy, and ILD is a recognized risk of treatment with CDK4/6 inhibitors [8]. These data underscore the need for treating physicians to consider the benefit–risk profile of abemaciclib when prescribing abemaciclib to patients.

Finally, 4 main imaging patterns were described for ILD in patients treated with abemaciclib, including OP, faint GGO, NSIP, and DAD patterns. Although no specific imaging patterns or lesion sites were identified, cases that presented with or developed DAD were observed more frequently in fatal cases. This is in accordance with previous reports of poorer outcomes for drug-induced ILD cases exhibiting DAD imaging features, including 40–83% mortality [5].

## Conclusion

Our results on potential risk factors contributing to the development of fatal cases of ILD in patients exposed to abemaciclib are of clinical importance, suggesting that advanced age and DAD in particular may be linked to poorer outcomes. However, it is difficult to conclude definitive risk factors based on this 1-year investigation, which was reliant on spontaneous reporting of adverse events by patients and HCPs. Specifically, prior to issuance of the Blue Letter, delays in seeking medical attention may have occurred due to reduced awareness of the risk of ILD following abemaciclib use and the failure to recognize and monitor for the symptoms of ILD. Therefore, confirmation of these potential risk factors awaits a higher level of evidence and further assessment. Despite these limitations, this study provides the first insight into the characteristics of ILD in patients treated with abemaciclib in the real-world setting in Japan and offers information for its detection, diagnosis, and treatment. HCPs should consider the benefit–risk balance when initiating patients on abemaciclib treatment, especially for those at a higher risk due to advanced age or ECOG PS, or pre-existing interstitial lesions. While on abemaciclib, patients should be regularly monitored for symptoms to ensure early detection of ILD. When ILD is suspected, imaging studies, such as CT, should be conducted as necessary, with discontinuation of abemaciclib as clinically indicated and treatment of ILD as per standard guidance.

## Supplementary Information

Below is the link to the electronic supplementary material.

Supplementary file1 (DOCX 14 KB)
