# Supplementary material for: Characteristics of interstitial lung disease in patients from post-marketing data on metastatic breast cancer patients who received abemaciclib in Japan
Source: Breast Cancer. 2021 Jan 16;28(3):710–9. doi: 10.1007/s12282-020-01207-8 (PMC8064939; doi:10.1007/s12282-020-01207-8)
Supplement: Supplementary file 1 — Supplementary file1 (DOCX 14 KB) [file 12282_2020_1207_MOESM1_ESM.docx]

**Supplementary Table 1. Terms from the SMQ for ILD used to identify potential ILD cases in the safety database**

| **Code** | **Preferred Term** |
| --- | --- |
| 10066728 | Acute interstitial pneumonitis |
| 10073344 | Alveolar lung disease |
| 10001881 | Alveolar proteinosis |
| 10001889 | Alveolitis |
| 10050343 | Alveolitis necrotising |
| 10080701 | Autoimmune lung disease |
| 10006448 | Bronchiolitis |
| 10076515 | Combined pulmonary fibrosis and emphysema |
| 10060902 | Diffuse alveolar damage |
| 10014952 | Eosinophilia myalgia syndrome |
| 10078117 | Eosinophilic granulomatosis with polyangiitis |
| 10014962 | Eosinophilic pneumonia |
| 10052832 | Eosinophilic pneumonia acute |
| 10052833 | Eosinophilic pneumonia chronic |
| 10081988 | Hypersensitivity pneumonitis |
| 10078268 | Idiopathic interstitial pneumonia |
| 10063725 | Idiopathic pneumonia syndrome |
| 10021240 | Idiopathic pulmonary fibrosis |
| 10082452 | Immune-mediated pneumonitis |
| 10022611 | Interstitial lung disease |
| 10025102 | Lung infiltration |
| 10070831 | Necrotising bronchiolitis |
| 10029888 | Obliterative bronchiolitis |
| 10035742 | Pneumonitis |
| 10036805 | Progressive massive fibrosis |
| 10037383 | Pulmonary fibrosis |
| 10058824 | Pulmonary necrosis |
| 10061473 | Pulmonary radiation injury |
| 10061924 | Pulmonary toxicity |
| 10037457 | Pulmonary vasculitis |
| 10037754 | Radiation alveolitis |
| 10037758 | Radiation fibrosis - lung |
| 10037765 | Radiation pneumonitis |
| 10080547 | Small airways disease |
| 10052235 | Transfusion-related acute lung injury |
| 10069351 | Acute lung injury |
| 10001052 | Acute respiratory distress syndrome |
| 10075289 | Airway remodelling |
| 10075185 | Allergic eosinophilia |
| 10068801 | Antisynthetase syndrome |
| 10004795 | Biopsy lung abnormal |
| 10010187 | Complications of transplanted lung |
| 10078811 | Cystic lung disease |
| 10018620 | Goodpasture's syndrome |
| 10072579 | Granulomatosis with polyangiitis |
| 10069152 | Granulomatous pneumonitis |
| 10069698 | Langerhans' cell histiocytosis |
| 10057261 | Lung induration |
| 10051604 | Lung transplant rejection |
| 10057481 | Lupus pneumonitis |
| 10049459 | Lymphangioleiomyomatosis |
| 10067472 | Organising pneumonia |
| 10035745 | Pneumonitis chemical |
| 10036024 | Polyarteritis nodosa |
| 10037313 | Pulmonary alveolar haemorrhage |
| 10037382 | Pulmonary eosinophilia |
| 10037391 | Pulmonary granuloma |
| 10037396 | Pulmonary haemosiderosis |
| 10068513 | Pulmonary renal syndrome |
| 10037430 | Pulmonary sarcoidosis |
| 10048667 | Restrictive pulmonary disease |
| 10039081 | Rheumatoid lung |
| 10039486 | Sarcoidosis |
| 10042954 | Systemic sclerosis pulmonary |
| 10051222 | Toxic oil syndrome |

ILD, interstitial lung disease; MedDRA, Medical Dictionary for Regulatory Activities; SMQ, standardized MedDRA query.
